# Gene profiling reveals association between altered Wnt signaling and loss of T-cell potential with age in human hematopoietic stem cells

**DOI:** 10.1111/acel.12229

**Published:** 2014-05-30

**Authors:** Melissa L M Khoo, Stephen M Carlin, Mark A Lutherborrow, Vivek Jayaswal, David D F Ma, John J Moore

**Affiliations:** 1 Blood Stem Cells and Cancer Research, St Vincent’s Centre for Applied Medical Research, and The University of New South WalesSydney, NSW, 2010, Australia; 2 Centre for Mathematical Biology, School of Mathematics and Statistics, University of SydneySydney, NSW, 2006, Australia; 3 School of Biomedical Sciences, Faculty of Health, Queensland University of TechnologyBrisbane, Qld, 4059, Australia

**Keywords:** aging, cellular immunology, gene expression, human, molecular biology of aging, mononuclear cells, T cell

## Abstract

Functional decline of the hematopoietic system occurs during aging and contributes to clinical consequences, including reduced competence of adaptive immunity and increased incidence of myeloid diseases. This has been linked to aging of the hematopoietic stem cell (HSC) compartment and has implications for clinical hematopoietic cell transplantation as prolonged periods of T-cell deficiency follow transplantation of adult mobilized peripheral blood (PB), the primary transplant source. Here, we examined the gene expression profiles of young and aged HSCs from human cord blood and adult mobilized PB, respectively, and found that Wnt signaling genes are differentially expressed between young and aged human HSCs, with less activation of Wnt signaling in aged HSCs. Utilizing the OP9-DL1 *in vitro* co-culture system to promote T-cell development under stable Notch signaling conditions, we found that Wnt signaling activity is important for T-lineage differentiation. Examination of Wnt signaling components and target gene activation in young and aged human HSCs during T-lineage differentiation revealed an association between reduced Wnt signal transduction, increasing age, and impaired or delayed T-cell differentiation. This defect in Wnt signal activation of aged HSCs appeared to occur in the early T-progenitor cell subset derived during *in vitro* T-lineage differentiation. Our results reveal that reduced Wnt signaling activity may play a role in the age-related intrinsic defects of aged HSCs and early hematopoietic progenitors and suggest that manipulation of this pathway could contribute to the end goal of improving T-cell generation and immune reconstitution following clinical transplantation.

## Introduction

Aging of the hematopoietic system has widespread effects on all cellular components, including hematopoietic stem cells (HSCs) and lymphoid progenitors, and causes progressive pathophysiological changes, such as the well-documented age-associated decline in lymphopoiesis (Linton & Dorshkind, 2004; Rossi *et al*., 2008). These perturbations culminate in clinically significant consequences, including reduced competence of adaptive immunity (Linton & Dorshkind, 2004) and increased incidence of myeloid diseases (Lichtman & Rowe, 2004). Age-related defects in the HSC compartment may also compound difficulties associated with allogeneic hematopoietic cell transplantation for the treatment of malignant and nonmalignant hematopoietic diseases, as prolonged periods of T-cell deficiency increase the risk of life-threatening complications (Mackall *et al*., 1995; Krenger *et al*., 2011).

Prior to clinical transplantation, preconditioning with chemotherapy and/or radiotherapy is required to reduce the presence of malignant cells, decrease the risk of graft rejection, and improve donor HSC engraftment. These regimens compromise immune function and predispose patients to infections, which may contribute to poor clinical outcomes. The primary unmet challenge of immune reconstitution is robust T-cell regeneration, as the majority of other hematopoietic cell types are restored within weeks (Krenger *et al*., 2011). T-lymphopoiesis is governed by seeding of the thymus with bone marrow (BM)-derived progenitors inherently capable of migration and T-lineage differentiation, as well as the ability of the thymus to direct this differentiation. It is well recognized that T-lymphocyte output decreases with age as a consequence of thymic involution. In humans, atrophy of the thymic epithelial space has been observed as early as 1 year after birth, although is most evident at puberty. Nevertheless, human adult thymic tissue remains capable of T-lymphocyte production at least until the sixth decade of life (Douek *et al*., 2000; Haynes *et al*., 2000).

Apart from age-induced stromal alterations, changes intrinsic to aged HSCs or lymphoid progenitors may also contribute to thymic involution and decreased T-lymphopoiesis. Indeed, it has been observed that BM or HSCs from old mice possessed less efficient T-cell generation when transplanted into young recipients (Tyan, 1977; Hirokawa *et al*., 1986; Sudo *et al*., 2000) or when cultured with fetal thymus explants (Sharp *et al*., 1990). More recently, these observations have been extended to humans, with our laboratory and others finding diverse *in vitro* T-cell potentials of hematopoietic progenitors originating from human prenatal (fetal thymus and liver), postnatal [cord blood (CB)], and adult (BM) tissues (Patel *et al*., 2009), as well as more rapid and extensive T-cell differentiation from CB-derived HSCs in comparison with adult-derived HSCs from BM (De Smedt *et al*., 2011; Carlin *et al*., 2012) and mobilized peripheral blood (PB) (De Smedt *et al*., 2011; Carlin *et al*., 2012). These findings are particularly pertinent to clinical hematopoietic transplantation, which currently utilizes adult mobilized PB as the main source of donor cells. A greater understanding of the effects of aging on HSCs may allow the development of strategies to improve T-cell generation from adult tissues and therefore improve immune reconstitution and survival post-transplantation.

Critical to T-lineage specification is the action of site-specific signals encountered within the thymic microenvironment that lead to activation of signaling pathways and downstream transcriptional programs. Involvement of Notch signaling is the most well understood and is known to be essential for inducing and supporting T-cell specification and commitment (Maillard *et al*., 2005; Petrie & Zuniga-Pflucker, 2007; Yuan *et al*., 2010). Following commitment, a decline in Notch activity occurs after the β-selection checkpoint in CD4^−^ CD8^−^ double-negative (DN) 3b thymocytes, suggesting that Notch signaling becomes dispensable for subsequent development (Schmitt *et al*., 2004; Taghon *et al*., 2005; Petrie & Zuniga-Pflucker, 2007; Yuan *et al*., 2010). T-cell differentiation from HSCs and progenitors can be achieved *in vitro* through co-culture with a BM stromal cell line (OP9) expressing high levels of Notch receptor ligand Delta-like 1 (DL1) (Schmitt & Zuniga-Pflucker, 2002). However, alternative pathways must be activated in conjunction with or in collaboration with Notch signaling, because T-cell differentiation induced by Notch ligand alone is blocked at the pre-T-cell stage (Reimann *et al*., 2012).

The canonical Wnt signaling pathway has also been implicated in hematopoiesis and T-cell development. Both *in vivo* and *in vitro* studies have demonstrated critical involvement of Wnt signal activation in T-lineage development (Verbeek *et al*., 1995; Staal *et al*., 2001; Mulroy *et al*., 2002), particularly the requirement of *TCF7* for differentiation, and the necessity of Wnt signaling at early DN stages (Weerkamp *et al*., 2006). However, conflicting findings on the role of Wnt in hematopoiesis have been reported in studies implementing forced Wnt activation or repression, which may be related to the lack of complete abolishment of Wnt signaling in these models (Jeannet *et al*., 2008; Luis *et al*., 2011). Further complexity exists, because both the Wnt and Notch signaling pathways are crucial to many cellular processes, and there is increasing evidence of cross talk between the two pathways (Munoz Descalzo & Martinez Arias, 2012), leaving the specific contribution of each to hematopoiesis and T-lineage development yet to be determined.

In this study, we examined the gene expression profiles of young and aged human HSCs from CB and adult mobilized PB, respectively, and found significant differential expression of genes in the Wnt signaling pathway. Utilizing the OP9-DL1 system to promote T-cell development under stable Notch signaling conditions, we found increased expression of β-catenin and Wnt target genes with T-cell differentiation. In addition, examination of Wnt signaling components and target gene activation in young and aged human HSCs revealed an association between reduced Wnt signal transduction, age, and impaired or delayed T-cell differentiation.

## Results

### Transcriptome profiling reveals differential gene expression in young and aged human HSCs

We have previously found that HSCs from adult PB have an intrinsic developmental block in T-lymphocyte differentiation compared with CB-derived HSCs (Carlin *et al*., 2012). To investigate the molecular mechanisms involved in the loss of T-lymphocyte potential with age, we isolated CD34^+^ HSC subsets (CD7^+/−^) from CB and adult PB by FACS and examined gene expression on a genome-wide scale by Affymetrix Gene Array analysis. More than 1300 genes were differentially expressed between young and aged HSCs (*P* < 0.05; CD7^−^ fraction: 1594 genes [480 of these genes were up-regulated > 1.5-fold; 836 genes were down-regulated > 1.5-fold); CD7^+^ fraction: 1392 genes (484 of these genes were up-regulated > 1.5-fold; 657 genes were down-regulated > 1.5-fold)].

**Figure 1:**
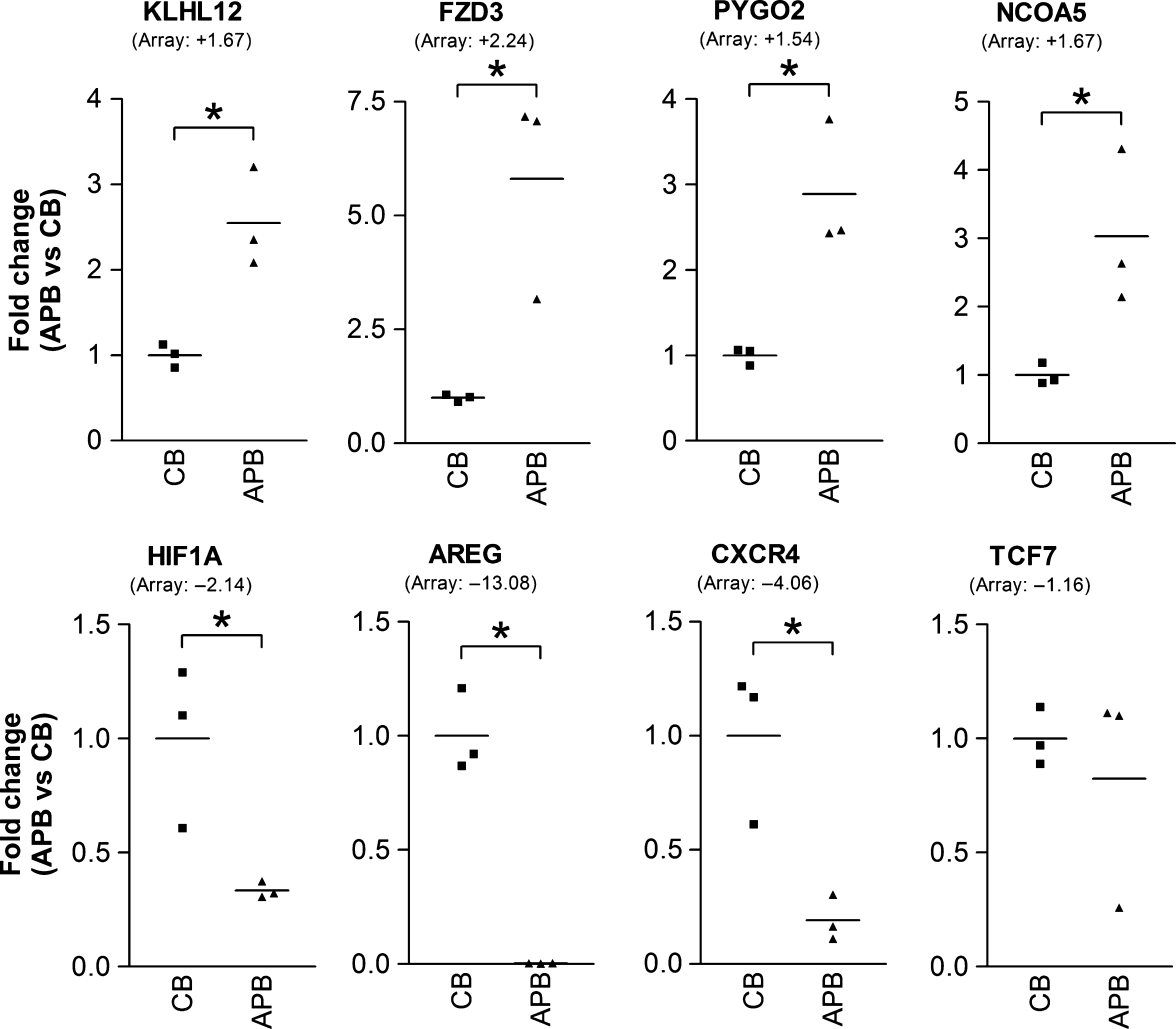
Real-time RT–PCR confirmation of Affymetrix array data. Verification of differential expression of Wnt signaling pathway genes, genes with low or high fold change, and genes with no significant change. Results are depicted as mean fold change in mRNA expression of adult mobilized peripheral blood HSCs (APB) relative to cord blood HSCs (CB). Array data are included within brackets for reference (also relative to CB). *P < 0.05 compared with CB.

We performed gene ontology enrichment analysis to determine whether the proportion of differentially expressed genes associated with a gene ontology term was higher than that obtained by chance. This enrichment analysis suggested that multiple signaling pathways were potentially dysregulated, including the Wnt signaling pathway (corrected *P*-values (collapse): ‘regulation of Wnt receptor signaling pathway’, *P* = 7.39 × 10^−8^; ‘Wnt receptor signaling pathway’, *P* = 2.32 × 10^−45^; ‘negative regulation of Wnt receptor signaling pathway’, *P* = 8.75 × 10^−6^; ‘positive regulation of Wnt receptor signaling pathway’, *P* = 0.175). This pathway was selected for further assessment, as our array data showed consistent regulation across this functional grouping (Table 1) and as Wnt signaling pathway components have been found to be involved in hematopoiesis and T-lineage development. The array data were verified by real-time RT–PCR (Fig. 1) to confirm the differential expression of Wnt signaling pathway genes, as well as genes with a low or high fold change and genes with no significant change. Good correlation was observed between the two techniques. In addition, the array data showed that genes for Wnt ligands, receptors, and inhibitors were expressed by both CB and adult PB HSCs, suggesting that both possess Wnt signaling capabilities (not shown; raw data in Table S1, Supporting information).

**Table 1:** Differential expression of genes involved in the Wnt signaling pathway in young and aged human HSCs by Affymetrix array

Young vs. aged	Fold change (CD7^+^/CD7^−^)	*P*-value (CD7^+^/CD7^−^)	Entrez ID	Name
Positive regulation of Wnt receptor signaling pathway				
*ZRANB1*	**1.73/1.87**	0.02/0.01	54764	Zinc finger, RAN-binding domain containing 1
*PPM1A*	**1.66/1.64**	0.04/0.04	5494	Protein phosphatase 1A (formerly 2C), magnesium-dependent, alpha isoform
Regulation of Wnt receptor signaling pathway				
*PPP2CA*	1.47/**1.80**	0.01/0.00	5515	Protein phosphatase 2 (formerly 2A), catalytic subunit, alpha isoform
*SENP2*	**1.94/2.10**	0.00/0.00	59343	SUMO1/sentrin/SMT3 specific peptidase 2
Negative regulation of Wnt receptor signaling pathway				
*CBY1*	**−1.97/−2.33**	0.02/0.01	25776	Chibby homolog 1 (Drosophila)
Wnt receptor signaling pathway				
*FZD3*	**−3.60/−2.24**	0.00/0.01	7976	Frizzled homolog 3 (Drosophila)

	Fold change (pos/neg)	*P*-value (pos/neg)	Entrez ID	Name
CD7^+^ (young vs. aged)				
Negative regulation of Wnt receptor signaling pathway				
*APC*	**−1.52**	0.05	324	Adenomatous polyposis coli
Wnt receptor signaling pathway				
*Ldb1*	**−1.62**	0.03	8861	LIM domain binding 1
*HBP1*	**1.79**	0.03	26959	HMG-box transcription factor 1
*Csnk1d*	1.37	0.03	1453	Casein kinase 1, delta
*Csnk1a1*	1.49	0.02	1452	Casein kinase 1, alpha 1
*LRP6*	**1.77**	0.04	4040	Low-density lipoprotein receptor-related protein 6
*FRAT1*	**−1.50**	0.02	10023	Frequently rearranged in advanced T-cell lymphomas
Wnt receptor signaling pathway through beta-catenin				
*APC*	**−1.52**	0.05	324	Adenomatous polyposis coli
CD7^−^ (young vs. aged)				
Wnt receptor signaling pathway				
*FBXW11*	1.49	0.02	23291	F-box and WD repeat domain containing 11
*Pygo2*	**−1.54**	0.02	90780	Pygopus homolog 2 (Drosophila)
*KLHL12*	**−1.67**	0.01	59349	Kelch-like 12 (Drosophila)

HSCs, hematopoietic stem cells. Fold change > 1.50 is shown in bold.

### Delayed expression of β-catenin in aged HSCs undergoing T-cell differentiation

**Figure 2:**
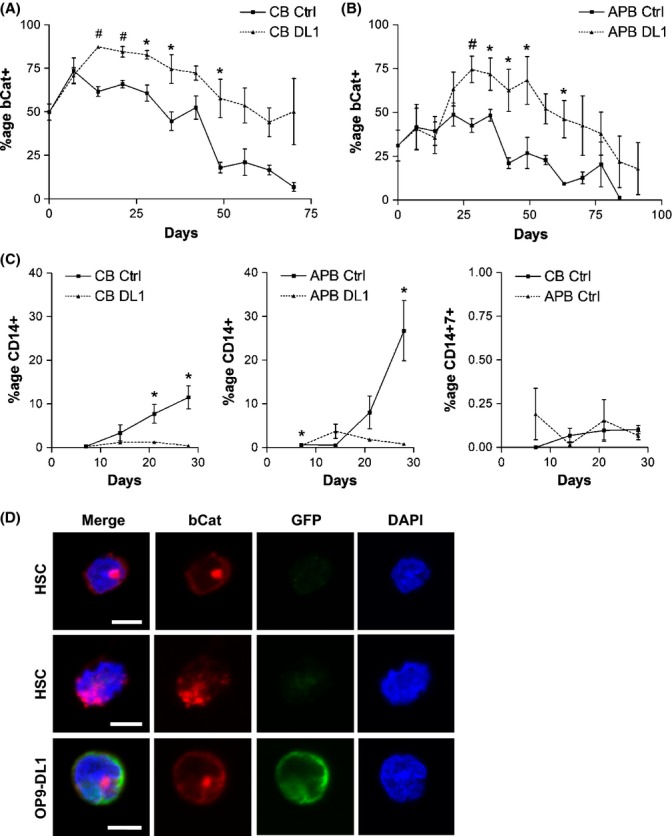
Increased expression of β-catenin in human hematopoietic stem cells (HSCs) cultured in T-cell differentiation conditions. Young (CB) and aged (APB) HSCs were co-cultured with OP9-Ctrl and OP9-DL1 stromal layers and analyzed by flow cytometry at weekly intervals. Percentage of β-catenin^+^ hematopoietic cells in co-cultures of (A) young HSCs (#*P* < 0.005, *P < 0.05; *n* = 3–5) and (B) aged HSCs (#*P* < 0.01, *P < 0.05; *n* = 3–7). (C) Percentage of CD14^+^ cells and CD14^+^ CD7^+^ cells generated in co-cultures of young and aged HSCs with OP9-Ctrl and OP9-DL1 stromal layers (*P < 0.05; *n* = 3). (D) Representative confocal sections depicting immunofluorescence staining of HSCs co-cultured with OP9-Ctrl and OP9-DL1 at different *z*-axis planes. Cells were examined for bCat (red) protein localization, and OP9 cells were identified by GFP (green) expression. Nuclei were stained with DAPI (blue). Nuclear expression of bCat was observed in young and aged HSCs during both myeloid and T-cell differentiation, and cytoplasmic bCat was also detected. Scale bar: 5 μm. CB: cord blood HSCs; APB: adult mobilized peripheral blood HSCs; bCat: β-catenin; DL1: OP9-DL1; Ctrl: OP9-Ctrl.

To examine the role of Wnt signaling in *in vitro* T-cell differentiation and the differences in Wnt activity between young and aged human HSCs, we co-cultured HSCs with OP9-DL1 cells and examined the levels of key Wnt signaling mediator β-catenin by intracellular flow cytometry over the course of long-term differentiation (2.5–3 months). Significantly higher levels of β-catenin protein were detected in HSCs cultured in T-lineage differentiation conditions compared with control for both CB and adult PB (Fig. 2A,B), indicating the presence of increased Wnt signaling activity during T-cell differentiation. It has been reported that adult HSCs produce myeloid- rather than T-lineage cells in OP9-DL1 co-culture (De Smedt *et al*., 2011); however, we only detected low levels (average < 2%) of myeloid marker CD14 in both adult PB and CB OP9-DL1 co-cultures (Fig. 2C), whereas control cultures yielded > 10% and 20% of CD14^+^ cells from CB and adult PB HSCs, respectively. Furthermore, CD14^+^ CD7^+^ cells were not detected (< 0.5%) in either adult PB or CB co-culture controls. Together, these findings suggest that increased Wnt signal activation is necessary during T-cell differentiation from human HSCs, whereas myeloid differentiation can proceed with moderate Wnt signal activation.

The cellular localization of β-catenin during myeloid and T-lineage differentiation was also examined by immunofluorescence staining and confocal microscopy (Fig. 2D). OP9 cells present in co-cultures were distinguished by staining with anti-GFP antibody. Both CB and adult PB co-cultures displayed nuclear staining for β-catenin under myeloid and T-lineage differentiation conditions. Cytoplasmic β-catenin staining was also observed. As β-catenin expression was not restricted to the cell membrane, and patterns of nuclear and cytoplasmic staining were observed, these findings support a role for β-catenin in transcriptional regulation and signal transduction, rather than cell adhesion alone.

**Figure 3:**
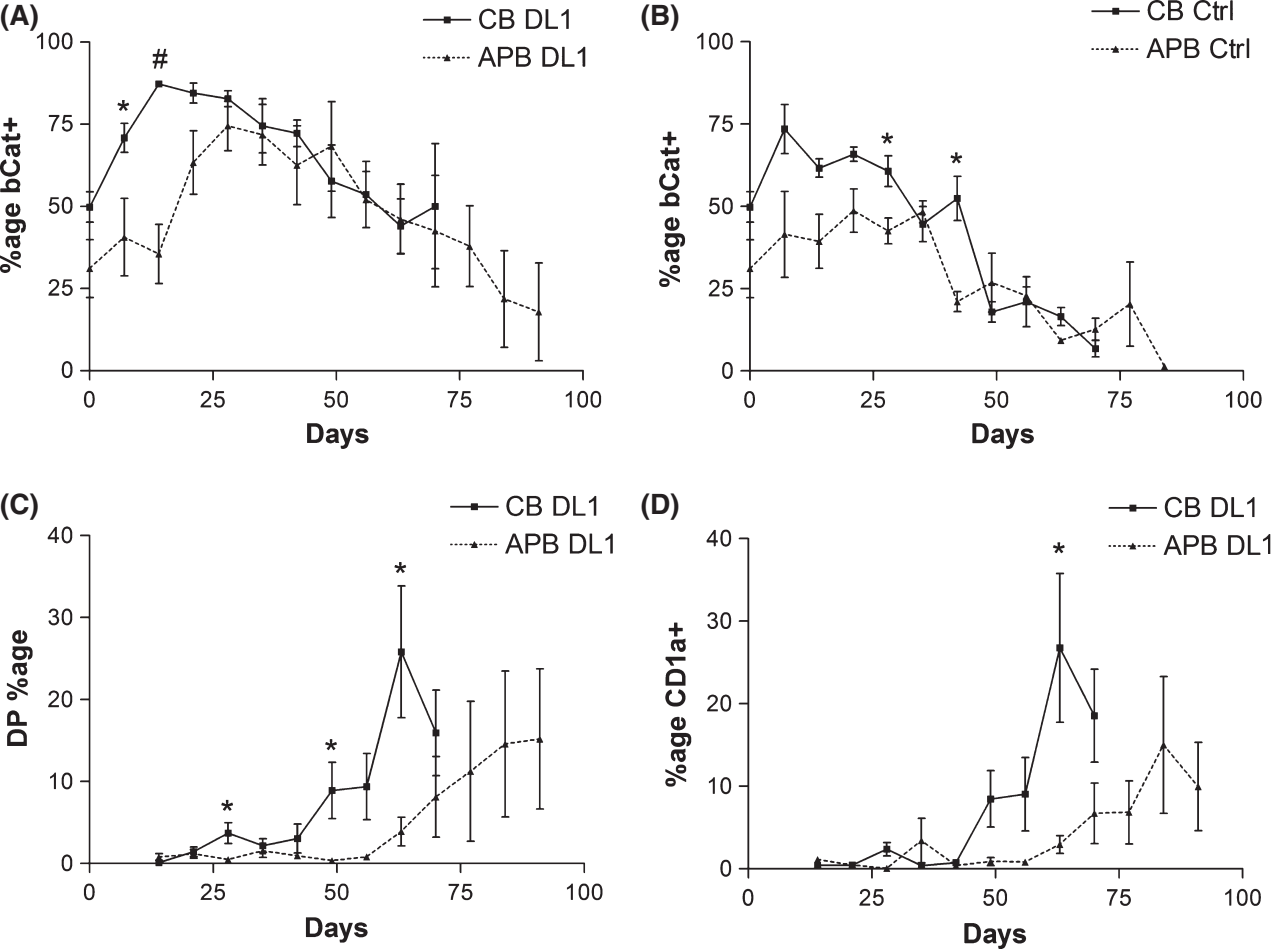
Aged human hematopoietic stem cells (HSCs) exhibit delayed β-catenin expression and DP generation compared with young HSCs during *in vitro* T-cell differentiation. Young (CB) and aged (APB) HSCs were co-cultured with OP9-Ctrl and OP9-DL1 stromal layers and analyzed by flow cytometry at weekly intervals. (A) Percentage of β-catenin^+^ hematopoietic cells in co-cultures of young and aged HSCs with OP9-DL1. Aged HSCs exhibit delayed β-catenin expression with significantly lower levels of β-catenin protein compared with young HSCs during the first few weeks of T-cell differentiation. (B) Percentage of β-catenin^+^ hematopoietic cells in co-cultures of young and aged HSCs with OP9-Ctrl. No difference in β-catenin expression level or timing in the first few weeks of co-culture in non-T-cell differentiation conditions. Generation of (C) DP cells and (D) CD1a^+^ cells from young and aged HSCs in T-cell differentiation conditions. APB: adult mobilized peripheral blood HSCs; CB: cord blood HSCs; bCat: β-catenin; DP: double-positive (CD4^+^ CD8^+^) T cell; DL1: OP9-DL1; Ctrl: OP9-Ctrl. *P < 0.05, #*P* < 0.001 (APB: *n* = 3–7, CB: *n* = 3–5).

Both young and aged HSCs up-regulated β-catenin during T-lineage differentiation; however, the timing of peak β-catenin expression differed. Cultures established with aged HSCs exhibited delayed β-catenin expression with significantly lower levels of β-catenin protein compared with young HSCs during the first 2–3 weeks of T-cell differentiation (Fig. 3A). This finding corresponds with our gene array data, with both suggesting that aged HSCs are less capable of Wnt signaling. In addition, the time frame of delayed β-catenin expression correlates with the delay in CD4^+^ CD8^+^ double-positive (DP) cell generation and CD1a expression observed in aged HSC co-cultures (Fig. 3C,D). No delay in CD14 generation was observed in control cultures of aged HSCs (Fig. 2C), and no difference was seen in β-catenin expression levels or timing (Fig. 3B); therefore, the defect in aged HSCs is specific to T-lineage differentiation and β-catenin expression.

### Wnt signaling defect is present in early T-progenitor cells derived from aged HSCs

**Figure 4:**
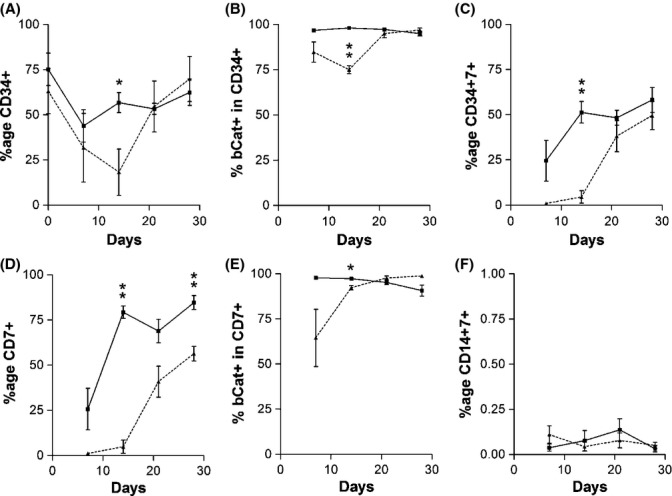
The Wnt signaling defect in aged human hematopoietic stem cells (HSCs) is present in the early T-progenitor subset. Young (solid line) and aged (dotted line) HSCs were co-cultured with OP9-DL1 stromal layers and examined for the expression of (A) CD34, (B) CD34 and β-catenin, (C) CD34 and CD7, (D) CD7, (E) CD7 and β-catenin, and (F) CD14 and CD7, during the first month of differentiation. β-Catenin is expressed by cells derived from both young and aged HSCs that possess characteristics of T-cell progenitors, but aged HSCs are initially fewer in proportion, limited in β-catenin expression, and exhibit delayed differentiation. bCat: β-catenin. *P < 0.05 (*n* = 3), **P < 0.01 (*n* = 3).

Our above findings show that early T-lineage differentiation of aged human HSCs is marked by reduced expression of β-catenin and impaired DP generation. To identify the cell subsets responsible for β-catenin expression in early T-cell differentiation, co-cultures derived from young and aged HSCs were examined for the expression of stem cell marker CD34 and T-cell progenitor marker CD7. In the first 2 weeks of OP9-DL1 co-culture, the percentage of CD34^+^ cells in aged HSC cultures decreases compared with young HSC cultures (*P* < 0.05 at day 14; Fig. 4A). Furthermore, there is significantly reduced β-catenin expression by aged CD34^+^ cells (*P* < 0.01; Fig. 4B). In contrast, young HSC cultures maintained CD34 expression (Fig. 4A), and all young CD34^+^ cells expressed β-catenin (Fig. 4B). Expression of CD7 increased gradually during the first month of differentiation in young HSC cultures, while aged HSCs were again delayed for the first 2 weeks (Fig. 4C,D). Interestingly, CD7 expression in aged HSCs occurred at day 21 (Fig. 4D), simultaneously with the point at which β-catenin expression in aged HSC cultures increased to reach similar levels as found in young HSC cultures (Fig. 3A). In addition, this also coincides with the increase in β-catenin expression by aged CD34^+^ cells (Fig. 4B). Finally, all CD7^+^ cells were found to express β-catenin, regardless of whether the cells were derived from young or aged HSCs (Fig. 4E), and CD14 was never found to be co-expressed with CD7 (Fig. 4F). These findings indicate that for both young and aged HSCs, β-catenin is expressed by cells that possess characteristics of T-cell progenitors; however, for aged HSCs, these T-cell progenitors are initially fewer in proportion, limited in β-catenin expression, and exhibit delayed differentiation. Impaired T-cell differentiation in aged HSCs may result from defects in Wnt signaling at early stages of differentiation, in cells that are still expressing CD34, but are in the process of gaining CD7.

### Differential expression of Wnt target genes by young and aged human HSCs

**Figure 5:**
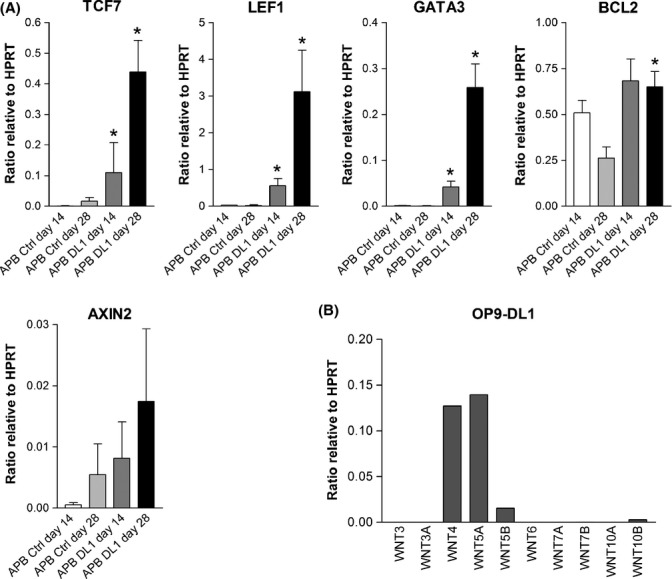
Real-time RT–PCR detection of Wnt target gene expression by human hematopoietic stem cells (HSCs) and Wnt gene expression by OP9-DL1 stromal layer. Results are depicted as mean expression ratio + SEM relative to reference gene *HPRT*. (A) Expression of Wnt target genes by aged (APB) HSCs undergoing T-cell differentiation in OP9-DL1 co-culture and in control co-cultures. Both young and aged HSCs exhibit greater Wnt transcriptional activity during T-lineage differentiation. *P < 0.05 (*n* = 3–4). (B) Wnt gene expression by OP9-DL1 cells. APB: adult mobilized peripheral blood HSCs; DL1: OP9-DL1; Ctrl: OP9-Ctrl; d: day.

To confirm that the observed differential β-catenin expression had functional implications on the Wnt transcriptional program, real-time RT–PCR for the detection of Wnt target gene expression was performed. Firstly, the expression of Wnt target genes, *TCF7*, *LEF1*, *GATA3*, and *BCL2*, was found to be significantly up-regulated in both young and aged human HSCs when cultured in OP9-DL1 differentiation conditions compared to control cultures (Fig. 5A). A trend of increasing expression was also observed for *AXIN2*. In addition, only a specific subset of Wnt target genes were up-regulated in OP9-DL1 co-culture (e.g., TCF7L1 was not differentially expressed and was only weakly expressed if at all, data not shown), suggesting that certain, but not all, Wnt target genes are involved in T-lineage differentiation. These findings of greater Wnt transcriptional activity in HSCs undergoing T-lineage differentiation are consistent with the increased levels of β-catenin seen by flow cytometry.

The OP9-DL1 co-culture system used for T-lineage differentiation is well known for stimulating Notch signaling. To determine whether Wnt signaling is also stimulated by this co-culture system, we examined OP9-DL1 cells for the expression of Wnt ligand genes that may be involved in activating Wnt signaling in HSCs during co-culture. OP9-DL1 stromal cells were found to express several Wnt genes, including *Wnt4* and *Wnt5A*, and to a lesser extent, *Wnt5B* and *Wnt10B* (Fig. 5B). Of these, Wnt4 has previously been reported to have a role in thymopoiesis (Staal *et al*., 2001; Mulroy *et al*., 2002). OP9-Ctrl cells showed a similar pattern of Wnt gene expression (data not shown).

**Figure 6:**
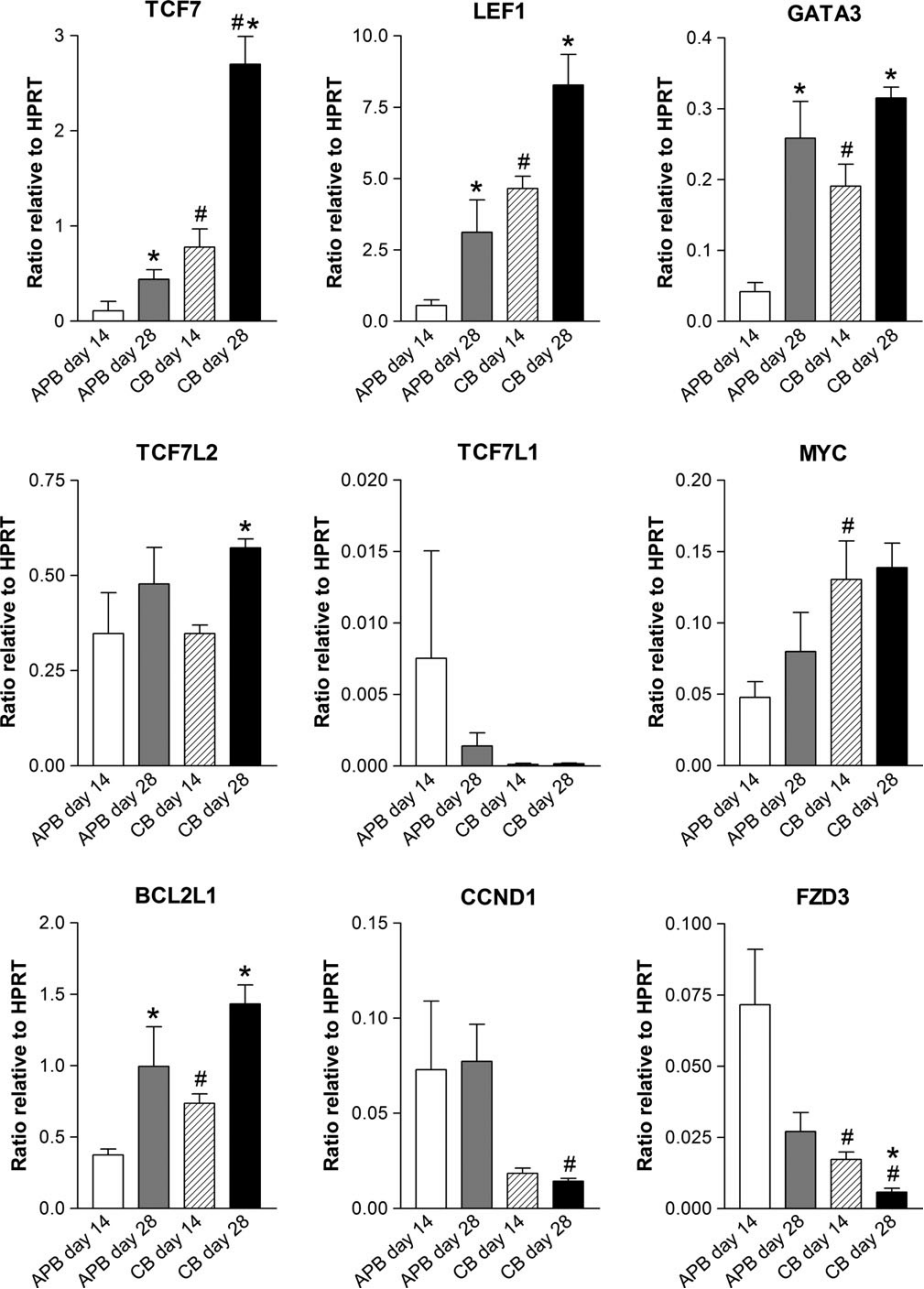
Differential expression of Wnt target genes by young and aged human hematopoietic stem cells (HSCs) following culture in T-cell differentiation conditions. Real-time RT–PCR results from young (CB) and aged (APB) HSCs co-cultured with OP9-DL1 stromal layer. Results are depicted as mean expression ratio + SEM relative to reference gene *HPRT*. Differential expression of Wnt target genes confirms the presence of differing levels of Wnt signaling in young and aged HSCs. *P < 0.05 d14 vs. d28 (*n* = 3–4), #*P* < 0.05 APB vs. CB (*n* = 3–4). APB: adult mobilized peripheral blood HSCs; CB: cord blood HSCs; d: day.

Next, the effect of the differential β-catenin expression in young and aged HSCs was examined in terms of Wnt target gene expression. Human HSCs undergoing T-lineage differentiation were assessed early during co-culture at days 14 and 28, because β-catenin was significantly reduced in aged HSCs at this period. Wnt target genes were found to be differentially expressed between young and aged HSCs during differentiation (Fig. 6; #*P* < 0.05 for young vs. aged HSCs). In particular, a subset of Wnt target genes, including *TCF7*, *LEF1*, *GATA3*, *MYC*, and *BCL2L1*, were significantly down-regulated in aged HSCs during T-lineage differentiation, providing further confirmation that aged HSCs are less capable of Wnt signaling. Several of these genes are also known to be involved in T-lineage differentiation (*TCF7*, *GATA3*, and *BCL2L1*), suggesting additional links between Wnt signaling, T-cell development, and aging. Not all Wnt target genes were down-regulated in aged HSCs, however, with *CCND1* more weakly expressed in young HSCs. In addition, *FZD3* receptor gene was significantly reduced in young HSCs, which is consistent with our array data, and suggests that at least some of the differential gene expression observed in the arrays was maintained by the cells during subsequent *in vitro* differentiation. Overall, these findings suggest that the different levels of Wnt signaling observed in young and aged HSCs, as inferred by the levels of β-catenin detected by flow cytometry, generate different effects on Wnt target gene expression that appear to influence T-lineage differentiation outcomes.

## Discussion

The ability to promote T-cell generation from mobilized adult PB could provide improved immune reconstitution following clinical transplantation and, as a result, improve patient outcomes. Increased understanding of age-related intrinsic defects in HSCs and early hematopoietic progenitors may contribute to this end goal. Here, we have shown that: (i) Wnt signaling genes are differentially expressed between young and aged human HSCs, with less activation of Wnt signaling in aged HSCs; (ii) Wnt signaling activity is important for T-lineage differentiation; (iii) reduced levels of Wnt signaling mediator β-catenin in aged HSCs are associated with delayed DP generation and differential Wnt target gene expression; and (iv) the defect in Wnt signal activation of aged HSCs occurs in the early T-progenitor cell subset derived during T-lineage differentiation.

Canonical Wnt signaling has previously been shown to play roles in the regulation of hematopoiesis and HSC function. In the past, a wide variety of approaches have been employed, and the resulting conflicting data have complicated the interpretation of these findings. More recently, murine models possessing different mutations in the adenomatous polyposis coli (*Apc*) gene have been generated that allow different gradients of Wnt signaling to be obtained *in vivo* (Luis *et al*., 2011). Utilizing this combination of hypomorphic alleles and conditional deletion alleles of *Apc*, it was demonstrated that differential activation of Wnt signaling occurs during hematopoiesis, with dosage-dependent effects determining self-renewal and differentiation to the myeloid and T-lymphoid lineages. Requirement of optimal Wnt dosages for different aspects of hematopoiesis provides an explanation for the variable results arising from different inducible gain-of-function approaches. In particular, it was found that enhanced T-cell development was only obtained with intermediate increases in Wnt signal activation, with early thymocyte subsets (DN) showing the greatest requirement for Wnt signaling. In contrast, maintenance of a multipotent HSC state was associated with mild increases in Wnt signal activation, while myeloid differentiation was only promoted by mild to intermediate increases in activation, and high to very high increases in Wnt activation were detrimental to murine HSC self-renewal and differentiation (Luis *et al*., 2011).

In the present study, we have found that different extents of Wnt signaling similarly occur in human HSCs, which appear to infer different lineage-specification abilities in an age-dependent manner. Aged human HSCs were found to possess intrinsic deficiencies in the expression of Wnt signaling mediators and Wnt target genes when compared with young human HSCs. Interestingly, the reduced activation of Wnt signaling in aged HSCs occurred together with the impairment of T-cell differentiation, while myeloid differentiation in control cultures was unaffected and no significant differences in β-catenin expression could be detected. Our data are consistent with the recent findings of lower requirements for Wnt signaling during myelopoiesis (Luis *et al*., 2011), suggesting that aged HSCs still possessed sufficient Wnt signaling capability for specification to the myeloid lineage, but insufficient levels for T-lymphocyte differentiation. In addition, this intrinsic defect in Wnt signaling of aged human HSCs may contribute to the reported myeloid-biased skewing of differentiation potential that is associated with aging of the hematopoietic system (Pang *et al*., 2011).

Interaction or cross talk between the Notch and Wnt signaling pathways has been reported with increasing frequency (Munoz Descalzo & Martinez Arias, 2012). Key transcriptional regulator of T-lineage development, TCF7, has classically been thought to be downstream of the Wnt signaling pathway. Recently, it was reported that activation of the Notch signaling pathway could also control the up-regulation of *TCF7* transcription, which was essential for progression through T-cell determination (Germar *et al*., 2011; Weber *et al*., 2011). In the present study, by transcriptional profiling of young and aged human HSCs, we only detected differential expression of a small number of Notch signaling-related genes (*PSEN1*, *NFKBIA*, *WDR12*). Furthermore, the pattern of expression was not consistent with reduced Notch signaling activity in aged HSCs. In addition, both young and aged human HSCs were exposed to similar stable Notch signaling conditions *in vitro* for the induction of T-cell differentiation. Our study suggests that the loss of T-cell potential in aged HSCs is linked to reduction in Wnt signaling activity; however, our data do not prove causality, and further studies are warranted to determine the contribution of other signaling pathways to the age-related functional deficits of HSCs. Given that some genes are common targets of both the Wnt and Notch signaling pathways, it is likely that a fine balance and integration of multiple signaling pathways must be maintained for T-lineage development and that this balance is upset during the aging process.

Our findings suggest that strategies targeting Wnt signaling activity in the early T-progenitor subset of aged human HSCs may improve immune reconstitution from these cells, through restoring Wnt activation to the levels found in young HSCs, which are necessary for T-lineage differentiation. A number of small-molecule pharmacologic activators of Wnt signaling [glycogen synthase kinase 3 (GSK3) inhibitors] have been developed, including BIO, CHIR99021, and TWS119; however, our preliminary investigations have suggested complexity in using these to activate Wnt signaling, potentially due to the difficulties in achieving the appropriate dosages for T-lineage differentiation. In support of this, it was recently shown that GSK3 inhibition with intermediate concentrations of CHIR99021 was capable of promoting DN3 differentiation *in vitro*, while higher concentrations suppressed DP development (Schroeder *et al*., 2013). On the other hand, potentiating Wnt signaling may be associated with negative effects, such as possible leukemogenesis (Lane *et al*., 2011).

Although targeting Wnt signaling would address the age-associated intrinsic deficits of HSCs, the existing thymic involution present in recipients may also have to be addressed to optimize T-cell regeneration. Despite increasing thymic atrophy with advancing age, the thymus maintains the capacity for thymopoiesis, as well as considerable regenerative potential (Haynes *et al*., 2000; Griffith *et al*., 2012). In recent years, strategies have been developed to enhance and repair thymic function through androgen blockade, administration of growth and differentiation factors, and transplantation of stem cell-derived thymic epithelial cells (Hollander *et al*., 2010; Lai *et al*., 2011). Apart from cell replacement approaches, these strategies rely on the regrowth of aged thymic tissue, which does transiently result in increased exportation of new T cells; however, while quantitative differences in cellularity are observed, qualitative changes in thymic stroma are absent and this may lead to a higher risk of self-reactivity in newly generated T cells (Griffith *et al*., 2012). Consistent with our findings, it was also shown that age-induced degeneration of thymic stroma was correlated with widespread changes in Wnt signaling that revealed significant transcriptional down-regulation with aging (Griffith *et al*., 2012). Therefore, enhancement of Wnt signal activation has the potential to provide twofold benefits to T-cell regeneration in an aged setting.

In conclusion, we have demonstrated that aged human HSCs possess reduced Wnt signaling activity in comparison with young HSCs, correlating with outcomes of impaired DP generation from aged HSCs, while myeloid differentiation was unaffected. Impaired T-cell differentiation from aged human HSCs is likely to be the result of defects in Wnt signaling in the early T-progenitor cell subset, suggesting that strategies aiming to enhance Wnt signal activation may be directed toward this subpopulation. Further investigations are warranted to allow a complete understanding of the multifactorial mechanisms underlying age-associated deficiencies in hematopoiesis to enable the development of new strategies for improving clinical immune reconstitution and T-lymphocyte regeneration.

## Experimental procedures

### Isolation of human HSCs from adult mobilized peripheral blood and cord blood

GCSF-mobilized PB from normal adult donors (21–57 years) was harvested by apheresis and cryopreserved in 10% DMSO, following written informed consent, as approved by the Human Research Ethics Committee of our Institute. Cells were thawed and washed with 1% BSA/PBS, and human HSCs were isolated as described (Carlin *et al*., 2012), using MACS lineage cell depletion kit (Miltenyi Biotec, Inc., San Diego, CA, USA) and FACS sorting (FACSAria II; BD Biosciences, San Jose, CA, USA) for selection of SSC^lo^CD45^mid^Lin^−^ CD34^+^ CD7^−^ and SSC^lo^CD45^mid^Lin^−^ CD34^+^ CD7^+^ subsets, after staining for CD45, CD34, and lineage markers (CD3, CD14, CD16, CD19, CD20, CD56) (all from BD Biosciences). Isolated cells were seeded onto a 24-well plate at 3 × 10^3^–10 × 10^3^ cells/well, over a monolayer of OP9 stromal cells, and co-cultures were maintained for up to 100 days.

Cord blood samples (> 36 weeks gestational age) were provided by the Sydney Cord Blood Bank after maternal consent, and mononuclear cells were separated on a Ficoll-Paque PLUS (GE Healthcare Bio-sciences AB, Uppsala, Sweden) density gradient (30 min at 400 *g*). Mononuclear cells were cryopreserved in 10% DMSO/20% fetal bovine serum (FBS)/PBS, and thawed cells were lineage-depleted and FACS sorted as described for adult mobilized PB.

### Gene expression profiling by Affymetrix microarray

Total RNA was obtained from FACS-isolated cell populations using RNeasy micro kit with DNase I treatment (Qiagen, Basel, Switzerland), according to the manufacturer’s instructions. Total RNA integrity and purity were assessed using Eukaryote Total RNA Nano or Pico chip on a Bioanalyzer (Agilent, Santa Clara, CA, USA). Only RNA with a RNA Integrity Number > 8.4 was used in the arrays. RNA was amplified using NuGEN Pico system (NuGEN Technologies, Inc., San Carlos, CA, USA) and then labeled and hybridized to genechip 1.0 ST arrays (Affymetrix; microarray service of The Ramaciotti Centre, UNSW). Individual samples were hybridized to separate array chips, without pooling of samples. Microarray data analysis was performed in R (R Core Team, 2012). First, the data were preprocessed using the Bioconductor package *affy* (Irizarry *et al*., 2003a). This involved RMA background correction (Irizarry *et al*., 2003b), quantile normalization (Bolstad *et al*., 2003), and summarization using the median polish algorithm. Second, the preprocessed data were analyzed using the Bioconductor package *limma* (Smyth, 2005) to test the null hypothesis that, for a given gene, the average expression was the same in the two conditions of interest. The two conditions of interest corresponded to young and aged HSCs in CD7^−^ or CD7^+^ fraction. Last, a gene was considered to be differentially expressed if the false discovery rate (Benjamini & Hochberg, 1995) adjusted *P*-value (for the null hypothesis) was < 0.05. A differentially expressed gene was considered to be up-regulated (resp. down-regulated) if the mean expression value for young HSC samples was higher (resp. lower) than that for aged HSC samples. Gene ontology analysis was performed using OntoExpress and DAVID Functional Annotation online tools.

### T-lineage differentiation in OP9-DL1 co-culture

The OP9 cell line stably expressing high levels of Notch receptor ligand Delta-like 1 (OP9-DL1) and GFP-expressing control cells (OP9-Ctrl) was obtained as a gift from Prof. Zuniga-Pflucker (Sunnybrook Research Institute, ON, Canada) via Prof. Gerard Hoyne (University of Notre Dame, WA, Australia). OP9 cells were maintained as described (Schmitt & Zuniga-Pflucker, 2002) in alpha-MEM containing 20% FBS and subcultured every 2–3 days by trypsinization. For co-culture with human HSCs, OP9 cells were seeded onto 24-well plates at 75% confluence. HSCs were added the next day in alpha-MEM supplemented with 20% FBS and cytokines, recombinant human SCF, Flt3-ligand, and IL-7 (all 5 ng/mL; R&D Systems, Inc., Minneapolis, MN, USA), and co-cultures were grown at 37 °C, 5% CO_2_, and 20% O_2_. Co-cultured hematopoietic progenitors were subcultured weekly onto fresh OP9 monolayers by vigorous pipetting and straining through 70-μm-mesh strainers. Aliquots of the resuspended cells were taken for analysis by flow cytometry, immunofluorescence microscopy, and real-time RT–PCR.

### Phenotypic characterization by flow cytometry

For characterization of surface antigen expression, co-cultured cells were stained with the following antibodies: CD34-PE-Cy7, CD7-PE, CD4-PE-Cy7, CD8-APC-Cy7, CD45-V450, CD14-V450, and CD3-PerCP-Cy5.5 (all from BD Biosciences). Cells were incubated with antibodies for 10 min, washed with PBS containing 0.2% NaN_3_ and 0.2% BSA, and resuspended in PBS with 0.5% paraformaldehyde. Multicolor analyses were performed using an LSRII flow cytometer (BD Biosciences), and data were analyzed using flowjo software (Tree Star, Inc., Ashland, OR, USA).

### Intracellular staining for flow cytometric analysis

For characterization of intracellular antigen expression, cells were fixed with cold 4% paraformaldehyde for 10 min and washed twice with PBS containing 0.2% NaN_3_ and 0.2% BSA. Cells were then permeabilized with BD FACS Permeabilizing Solution 2 (BD Biosciences) for 10 min, washed, incubated for 30 min with anti-human β-catenin-APC monoclonal antibody (R&D Systems, Inc.), washed, resuspended in PBS with 0.5% paraformaldehyde, and analyzed as above.

### Immunofluorescence staining and analysis

Co-cultured cells in suspension were fixed and permeabilized as above. Normal goat serum (10%) was applied for 1 h at room temperature to block cells, followed by incubation with primary antibodies at room temperature for 1 h. Primary antibodies included mouse monoclonal IgG anti-human β-catenin-APC (R&D Systems, Inc.) and rabbit polyclonal anti-GFP (Abcam, Inc., Cambridge, UK). Cells were washed twice and incubated with Alexa Fluor 594 goat anti-mouse IgG and Alexa Fluor 488 goat anti-rabbit secondary antibodies (1:400; Molecular Probes, Life Technologies, Carlsbad, CA, USA) for 2 h. Negative controls consisted of secondary antibody application alone. Cells were washed twice, and nuclei were stained with 300 nm DAPI (Molecular Probes, Life Technologies) in PBS for 10 min, followed by a final wash and mounting in 100% glycerol. Confocal imaging was performed using a Leica laser-scanning confocal microscope (DMI 6000 SP8; Leica Microsystems, Wetzlar, Germany).

### RNA extraction and real-time RT–PCR

Total RNA was extracted using TRIzol reagent (Life Technologies) and RNeasy micro kit with DNase I treatment (Qiagen). Total RNA was reverse-transcribed with Superscript III (Life Technologies) using 25 ng random hexamers and 2.5 μm oligo(dT)_20_ primers, according to the manufacturer’s recommendations and with RNase H treatment. Real-time RT–PCR was performed as previously described (Carlin *et al*., 2012), using Platinum SYBR Green qPCR SuperMix-UDG (Life Technologies) on a Rotor-Gene RG3000 machine (Corbett Research, Sydney, NSW, Australia). Primers are listed in Table 2.

**Table 2:** Primer sequences for real-time RT–PCR

Gene	GenBank number	Sequence (5′–3′)		Product size (bp)
		Sense	Antisense	
*KLHL12*	NM_021633	aatgcagggatctggttgat	gagactgctggcttccaaag	144
*FZD3*	NM_017412	ccttgaggatgtgccaagat	gaaatcccgagaacaatcca	137
*PYGO2*	NM_138300	tccagaaaagaagcgaagga	tcttcaaaagggttggatgc	120
*NCOA5*	NM_020967	atccagagacgctttgatgc	acctcccctgctaacatcct	186
*HIF1A*	NM_001530	tcatccaagaagccctaacg	tccatttttcgctttctctga	120
*AREG*	NM_001657	gggagtgagatttcccctgt	tcactttccgtcttgttttgg	176
*CXCR4*	NM_001008540	gaagctgttggctgaaaagg	ctcactgacgttggcaaaga	94
*TCF7*	NM_003202	agccagaagcaagttcacagg	gcctccttctctgccttggac	257
*LEF1*	NM_001130713	gatcacacccgtcacacatc	acccggagacaagggataaa	104
*GATA3*		agccactcctacatggacgc	aaggggctgagattccaggg	257
*BCL2*	NM_000633	gagtacctgaaccggcacct	tcacttgtggcccagatagg	186
*AXIN2*	NM_004655	aagctgaagctggagttgga	cagtatcgtctgcgggtctt	180
*TCF7L2*	NM_001146274	gctgagtgcacgttgaaaga	tttcgcttgctcttctctgg	87
*TCF7L1*	NM_031283	ctttaaaggacccccgtacc	tgacctcgtgtccttgactg	169
*MYC*	NM_002467	ctggtgctccatgaggaga	actctgaccttttgccagga	124
*BCL2L1*	NM_138578	cagcttggatggccacttac	tgctgcattgttcccataga	101
*CCND1*	NM_053056	ccctcggtgtcctacttcaa	ctcctcgcacttctgttcct	108
*WNT3*†	NM_009521.2	atctttgggcctgtcttgg	tggccccttatgatgtgagt	147
*WNT3A*†	NM_009522.2	ccatctttggccctgttct	tcactgcgaaagctactcca	87
*WNT4*†	NM_009523.1	actggactccctccctgtct	gtcacagccacacttctcca	145
*WNT5A*†	NM_009524.2	aggagttcgtggacgctaga	aggctacatctgccaggttg	132
*WNT5B*†	NM_009525.3	ccgagagcgtgagaagaact	ggcgacatcagccatcttat	115
*WNT6*†	NM_009526.3	cggaagtagtggcagagctt	ggaaacggaactggaactga	72
*WNT7A*†	NM_009527.3	ccgggagatcaagcagaat	tggtccagcacgtcttagtg	143
*WNT7B*†	NM_009528.3	tcgacttttctcgtcgcttt	cctgacacaccgtgacactt	142
*WNT10A*†	NM_009518.2	gactccacaacaaccgtgtg	cctactgtgcggaactcagg	133
*WNT10B*†	NM_011718.2	ttctctcgggatttcttgga	cacttccgcttcaggttttc	116
Ubiquitous markers				
*HPRT1*	NM_000194	gaccagtcaacaggggacat	cctgaccaaggaaagcaaag	132
*GAPDH*	NM_002046	aatcccatcaccatcttcca	tggactccacgacgtactca	82
*mHPRT*†	NM_013556.2	gccgaggatttggaaaaagt	aatccagcaggtcagcaaag	157
*ACTB*†	NM_007393	tgttaccaactgggacgaca	ggggtgttgaaggtctcaaa	165

† Primers used to detect gene expression in mouse cells (all other primers are for human cells).

### Statistical analysis

PCR confirmation of Affymetrix array data was expressed as mean fold change in CB expression relative to APB expression. Time-course flow cytometry data were expressed as mean percentage expression + SEM. Gene expression data were expressed as mean fold change in mRNA expression + SEM relative to control cultures, or mean expression ratio + SEM relative to reference gene *HPRT*. Significance levels were determined by *t*-test analysis (*P* < 0.05). Graphs were produced using graphpad prism 3.0 (GraphPad Software, Inc., La Jolla, CA, USA).

## Supporting Information

**Table S1** Raw complete microarray data sets.
