# Supplementary material for: Gene profiling reveals association between altered Wnt signaling and loss of T-cell potential with age in human hematopoietic stem cells
Source: Aging Cell. 2014 May 30;13(4):744–54. doi: 10.1111/acel.12229 (PMC4326953; doi:10.1111/acel.12229)
Supplement: Supplementary file 1 — Table S1 Raw complete microarray data sets. [file acel0013-0744-sd1.docx]

**Table S1** Raw complete microarray data sets.
